# *In utero* TNF‐α treatment induces telomere shortening in young adult mice in an ATF7‐dependent manner

**DOI:** 10.1002/2211-5463.12006

**Published:** 2016-01-04

**Authors:** Binbin Liu, Toshio Maekawa, Bruno Chatton, Shunsuke Ishii

**Affiliations:** ^1^ Laboratory of Molecular GeneticsRIKEN Tsukuba InstituteIbarakiJapan; ^2^ Graduate School of Comprehensive Human SciencesUniversity of TsukubaIbarakiJapan; ^3^ UMR7242 Biotechnologie et Signalisation CellulaireUniversité de StrasbourgIllkirchFrance

**Keywords:** activating transcription factor‐7, maintenance, stress, telomere shortening, tissue specificity, tumour necrosis factor‐α

## Abstract

Epidemiological studies indicate that exposure to stress during intrauterine life is associated with shorter telomeres in young adulthood, and a correlation between telomere length in early life and lifespan has been suggested. However, empirical studies evaluating these phenomena have not been performed, and the mechanism of stress‐induced telomere shortening remains unknown. Since the level of tumour necrosis factor α (TNF‐α) in peripheral blood cells is increased by various psychological stresses, the effect of TNF‐α administration to pregnant mice on telomere length in adulthood was examined in the present study. *In utero* TNF‐α treatment‐induced telomere shortening in adult mice. Telomere shortening was observed in certain tissues such as the bone marrow, spleen, and lung, and was detected at specific age ranges during adulthood. Telomere shortening was not observed in mice lacking the stress‐responsive transcription factor ATF7, which contributes to heterochromatin formation in the absence of stress. The present study identified the conditions under which *in utero* TNF‐α treatment induces telomere shortening in adulthood.

**Abbreviations**
ATF: activating transcription factor
ChIP: chromatin immunoprecipitation
dATF2: *Drosophila* ATF2
ROS: reactive oxygen species
TNF‐α: tumour necrosis factor‐α
WT: wild‐type

Telomeres consist of tandem TTAGGG repeats and maintain the integrity of chromosome ends during cell division 1, 2. In most somatic cells, telomeres shorten with cell division and generally with aging 3. Telomere shortening is associated with various diseases, including cancers and dyskeratosis congenita 4, as well as changes in cellular metabolism 5. Epidemiological studies suggest that telomere shortening is associated with a wide range of stress‐inducing life situations, and it has been detected in caregivers of chronically ill children 6, women exposed to partner violence 7, and patients with stress‐related mood disorders 8, 9. Maternal exposure to psychosocial stresses during pregnancy, including the death or sudden severe illness of an immediate family member or loss of primary residence, is associated with shorter telomeres in the offspring in adult life 10. However, because of the lack of empirical studies addressing these phenomena, many questions remain unanswered, such as the duration of stress‐induced telomere shortening and whether it affects all types of cells.

Activating transcription factor‐7 (ATF7) is a vertebrate member of the ATF2 subfamily of transcription factors, which belong to the ATF/CREB superfamily of proteins. The ATF2 subfamily of transcription factors is characterized by the presence of phosphorylation sites for stress‐activated protein kinase p38 and b‐ZIP DNA‐binding domains 11, 12, 13. Environmental, oxidative, psychological, and nutritional stresses, as well as inflammatory cytokines, such as tumour necrosis factor‐α (TNF‐α), and pathogen infection, induce the p38‐mediated phosphorylation of the ATF2 subfamily of transcription factors, including ATF7 14, 15. In the absence of stress, ATF7 silences a group of target genes, including the serotonin receptor 5b gene in the brain and innate immune genes in macrophages, by recruiting the ESET/SET‐DB1 histone H3K9 trimethyltransferase and G9a histone H3K9 dimethyltransferase, respectively, leading to heterochromatin formation 16, 17. Social isolation, a kind of psychological stress, and pathogen infection induce ATF7 phosphorylation by p38 in the brain and macrophages, respectively, resulting in the release of ATF7 and ESET or G9a from their target genes, leading to transcriptional activation and the long‐term maintenance of high basal expression levels. *Drosophila* ATF2 (dATF2) and yeast *S*. *pombe* Atf1, which are orthologs of ATF7, contribute to heterochromatin formation 18, 19. Environmental stresses, such as heat shock or osmotic stress, induce dATF2 phosphorylation and the release of dATF2 from heterochromatic structures, leading to an inheritable disruption of heterochromatin 18.

In this study, the association between maternal psychological stress exposure during pregnancy and telomere shortening in adult life was assessed using TNF‐α, which is induced in peripheral blood cells by various psychological stresses 20. The present results showed that TNF‐α exposure during pregnancy induced telomere shortening in certain tissues such as the bone marrow, spleen, and lung during a specific age range in adulthood in an ATF7‐dependent manner.

## Materials and methods

### Mice

Congenic wild‐type (WT) and *Atf7* ^*−/−*^C57BL/6 mice, which were described previously 16, were used. Experiments were performed in accordance with the guidelines of the Animal Care and Use Committee of the RIKEN Institute.

### TNF‐α administration

TNF‐α (10 or 20 μg·kg^−1^ weight) was intraperitoneally administered to pregnant mice daily between E2.5 and E18.5. DNA was prepared from blood cells or various tissues of 1–6‐week‐old male mice, and used for Q‐PCR to measure telomere length.

### Preparation of DNA from various tissues

Different tissues were isolated from mice at different times, and DNA was isolated using a DNeasy Blood & Tissue Kit (Qiagen, Hilden, Germany). For blood samples, a red blood cell lysis buffer (0.155 m NH_4_Cl, 0.01 m KHCO_3_, and 0.1 mm EDTA, pH 7.4) was used to remove red blood cells.

### Measurement of telomere length by Q‐PCR

Using a real‐time quantitative PCR method previously described 21, average telomere length was measured in total genomic DNA prepared from various tissues. In this assay, the average telomere length ratio is determined by quantifying telomeric DNA using a specifically designed primer sequence, and then dividing that value by the level of a single‐copy gene measured in the same sample (36B4). The primer sequences used are shown in Table S1. Real‐time PCR was performed using 1.5 ng DNA as a template in an ABI 7500 real‐time PCR instrument with SYBR Green PCR Master Mix (Applied Biosystems, Warrington, UK), as previously described 21, 22. The PCR conditions for telomeres were as follows: 95 °C for 10 min, 25 cycles of 95 °C for 15 s, and 54 °C for 2 min. The PCR conditions for 36B4 were as follows: 95 °C for 10 min, 25 cycles of 95 °C for 15 s, 52 °C for 20 s and 72 °C for 32 s. For each PCR reaction, a standard curve was prepared using serial dilutions of known amounts of DNA. The telomere signal (T) was normalized to the signal from the single‐copy gene 36B4 (S) to generate a T/S ratio, indicative of relative telomere length. Equal amounts of DNA were used for each reaction, and several replicates of each reaction were run.

### Chromatin immunoprecipitation/slot‐blot hybridization

Chromatin immunoprecipitation (ChIP) assays were carried out as described previously 17. Splenocytes were crosslinked in 0.5% formaldehyde for 8 min at room temperature, and glycine was added to a final concentration of 0.125 m to quench the crosslinking reaction. The chromatin was solubilized and extracted with lysis buffer (50 mm Tris‐HCl, pH 8.0, 10 mm EDTA, 1% SDS, and protease inhibitor cocktail). The DNA was sheared into approximately 500 bp fragments by sonication and diluted 10‐fold with dilution buffer (10 mm Tris–HCl, pH 8.0, 100 mm NaCl, 1 mm EDTA, 0.5 mm EGTA, 1% Triton X‐100 and 0.1% deoxycholate). Immunoprecipitation was performed overnight at 4 °C with anti‐ATF7 (2F10), anti‐H3 (ab1791; Abcam, Cambridge, UK) and anti‐H3K9me3 (ab8898; Abcam) antibodies. Normal anti‐mouse IgG antibodies were used as controls. Immunocomplexes were recovered using Dynabeads® Protein G beads (10009D; Life Technologies, Oslo, Norway), and then incubated at 65 °C in 130 μL of IP elution buffer (1% SDS, 0.1 m NaHCO_3_, 250 mm NaCl, 200 mg·mL^−1^ proteinase K, and 10 mm DTT) to release proteins. The DNA was further purified using a QIAquick PCR Purification Kit (Qiagen) and eluted in 100 μL of elution buffer. Eluted DNA samples were blotted onto Biodyne B membranes. Membranes were hybridized overnight in hybridization buffer containing 7% sodium dodecyl sulphate, 1% BSA, 0.5 m phosphate buffer (pH7.2), and 1 mm EDTA at 42 °C with the following ^32^P‐labelled oligonucleotide telomeric probes: G24 probe, [TTAGGG]_4_; C24 probe, [CCCTAA]_4_. Signals were visualized and quantified using Image Analyzer 2500 (Fuji, Tokyo, Japan).

### Statistics

Results are presented as means ± standard deviation (SD). The statistical significance of differences between groups was determined with the Student's *t*‐test.

## Results

### *In utero* TNF‐α treatment induces telomere shortening in the blood cells of infant and young adult mice

**Figure 1:**
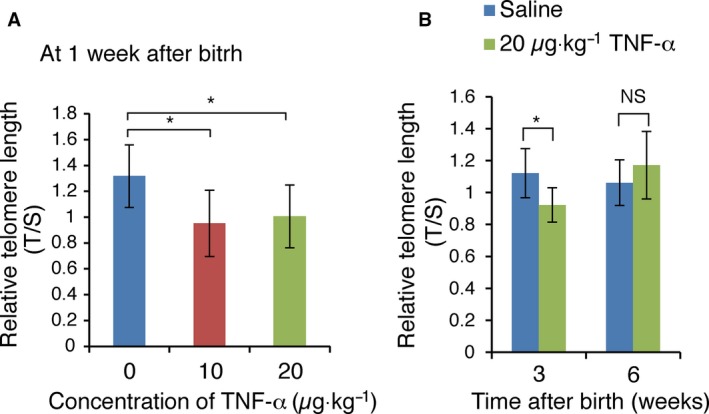
*In utero* TNF‐α treatment induces telomere shortening in the blood cells of infant and young adult mice. (A) Wild‐type (WT) pregnant mice were intraperitoneally injected with saline or TNF‐α (10 or 20 μg·kg^−1^ weight) daily from E2.5 to E18.5. Blood cells were prepared from 1‐week‐old mice, and used for telomere length measurement by Q‐PCR. (B) WT pregnant mice were intraperitoneally injected with saline or TNF‐α (20 μg·kg^−1^ weight) daily from E2.5 to E18.5. Blood cells were prepared from 3‐ or 6‐week‐old mice, and used for telomere length measurement by Q‐PCR. Relative telomere length, expressed as T/S ratio (see Materials and methods), is shown ± SD (*n* = 6–8). *P <* *0.05; N.S., no significant difference.

The effect of TNF‐α injection into pregnant mice on telomere length in the young adult was tested. We selected the TNF‐α concentration, 10 or 20 μg·kg^−1^, which induces inflammatory process but not developmental defect, because we used TNF‐α treatment as the method which mimics psychological stress. Injection of TNF‐α into mice was previously shown to induce the expression of multiple genes 23. Therefore, we examined gene expression levels and showed that *Cxcl10, Cxcl9, NF‐*κ*B p65* and *Cd14* were significantly induced in the liver in response to administration of 20 μg·kg^−1^ of TNF‐α to pregnant mice (data not shown). Pregnant mice received daily intraperitoneal injections of 10 or 20 μg·kg^−1^ of TNF‐α or saline from E2.5 to E18.5, and DNA was prepared from the blood cells of newborn mice at 1 week after birth. Assessment of telomere length by Q‐PCR showed telomere shortening in the blood cells of 1‐week‐old mice exposed to 10 or 20 μg·kg^−1^ of TNF‐α from E2.5 to E18.5 (Fig. 1A). In 3‐week‐old mice born to mothers treated with 20 μg·kg^−1^ of TNF‐α from E2.5 to E18.5, blood cells showed similar telomere shortening (Fig. 1B), which was not observed in the blood cells of 6‐week‐old mice. These results indicated that *in utero* TNF‐α treatment‐induced telomere shortening in the blood cells of 1‐ and 3‐week‐old mice, but not in 6‐week‐old mice.

### *In utero* TNF‐α treatment induces telomere shortening in some adult tissues

**Figure 2:**
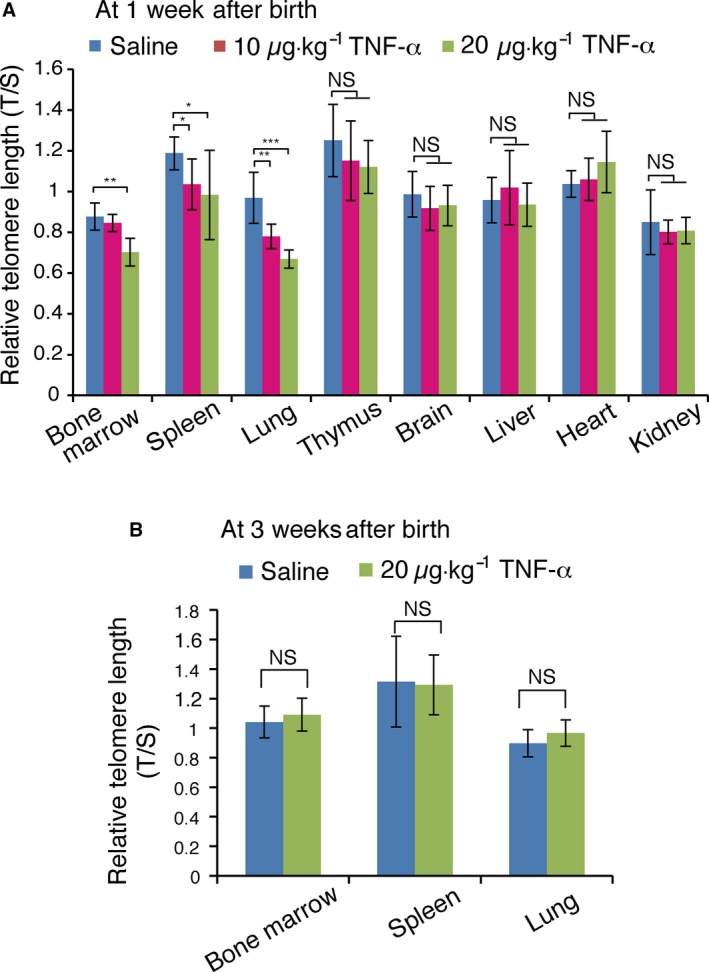
*In utero* TNF‐α treatment induces telomere shortening in specific tissues of infant mice. (A and B) WT pregnant mice were intraperitoneally injected with saline or TNF‐α (10 or 20 μg·kg^−1^ weight) daily from E2.5 to E14.5. DNA was prepared from the indicated tissues of 1‐week‐old (A) or 3‐week‐old (B) mice, and used for telomere length measurement by Q‐PCR. Relative telomere length, expressed as T/S ratio (see Materials and methods), is shown ± SD (*n* = 6–8). *P <* *0.05; **P <* *0.01; ***P < 0.001; N.S., no significant difference.

Assessment of telomere length in humans is limited to blood cells. We therefore used 1‐week‐old mice exposed to *in utero* TNF‐α treatment to measure telomere length in different tissues. Pregnant mice were treated with 10 or 20 μg·kg^−1^ TNF‐α from E2.5 to E18.5, which resulted in telomere shortening in the bone marrow of 1‐week‐old mice exposed to 20 μg·kg^−1^, but not in those exposed to 10 μg·kg^−1^ TNF‐α (Fig. 2A). In the spleen and lungs, telomere shortening was detected with both 10 and 20 μg·kg^−1^ of TNF‐α treatment *in utero* (Fig. 2A). However, telomere shortening was not observed in the thymus, brain, heart, liver, and kidney of 1‐week‐old mice. These results indicated that *in utero* TNF‐α treatment‐induced telomere shortening in the bone marrow, spleen, and lungs at 1 week after birth.

Next, telomere shortening induced by *in utero* TNF‐α treatment was examined at 3 weeks after birth. Injection of 20 μg·kg^−1^ TNF‐α into pregnant mice did not cause telomere shortening in the bone marrow, spleen and lungs of 3 week‐old mice (Fig. 2B). These results indicated that *in utero* TNF‐α treatment‐induced telomere shortening of certain types of cells, including blood cells, bone marrow, spleen, and lungs of infant 1‐week‐old mice, whereas in 3‐week‐old mice, it was no longer detected in most tissues, and was only retained in blood cells.

### *In utero* TNF‐α treatment does not induce telomere shortening in Atf7‐deficient mice

**Figure 3:**
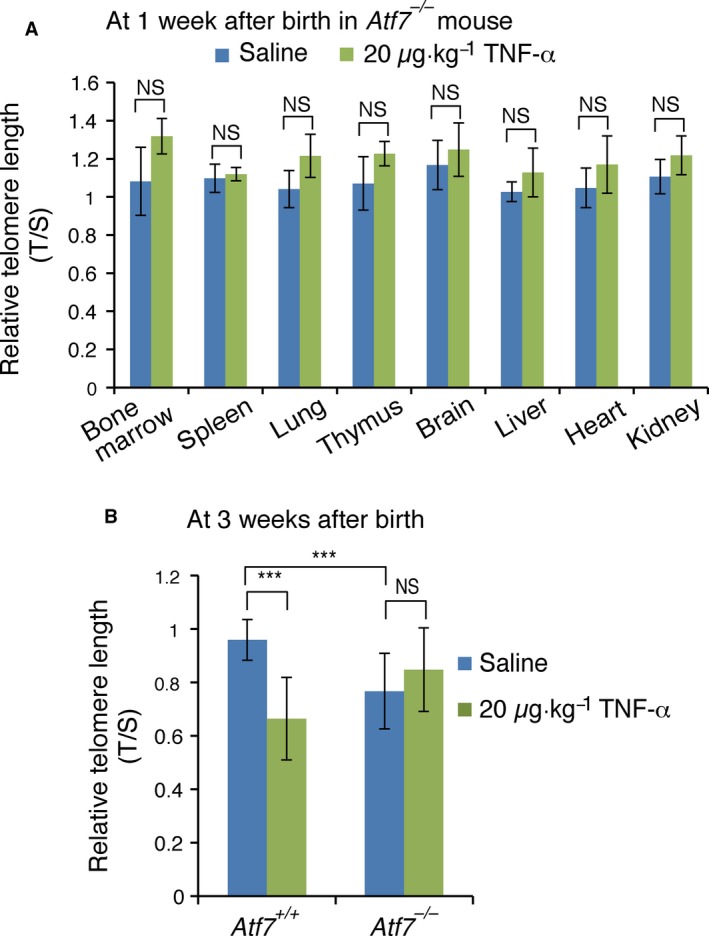
*In utero* TNF‐α treatment does not induce telomere shortening in *Atf7*‐deficient mice. (A and B) WT or *Atf7* deficient (*Atf7* ^*−/−*^) pregnant mice were intraperitoneally injected with saline or TNF‐α (20 μg·kg^−1^ weight) daily from E2.5 to E18.5. Since *Atf7* ^*−/−*^ pregnant mice were generated by mating with *Atf7* ^*−/−*^ male mice, all mice born were *Atf7* ^*−/−*^ mice. DNA was isolated from the indicated tissues of 1‐week‐old mice (A) or the blood cells of 3‐week‐old mice (B), and used for telomere length measurement by Q‐PCR. Relative telomere length, expressed as T/S ratio (see Materials and methods), is shown ± SD (*n* = 3–4). ***P <* *0.001; N.S., no significant difference.

The stress‐responsive transcription factor ATF7 contributes to the formation of a heterochromatin‐like structure by recruiting the ESET/SET‐DB1 histone H3K9 trimethyltransferase and G9a histone H3K9 dimethyltransferase 16, 17. In addition, ATF7 mediates the formation of pericentromeric heterochromatin by recruiting the Suv39 h1 histone H3k9 trimethyltransferase(K. Yoshida, T. Maekawa, Y. Zhu, C. Renard‐Guillet, B. Chatton, K. Inoue, T. Uchiyama, K. Ishibashi, T. Yamada, N. Ohno, K. Shirahige, M. Okada‐Hatakeyama & S. Ishii, unpublished). TNF‐α induces ATF7 phosphorylation by p38 16, and ATF7 phosphorylation results in disruption of the heterochromatin‐like structure 16, 17. To examine whether the *in utero* TNF‐α‐induced telomere shortening in blood cells of young adult mice is dependent on ATF7, *Atf7‐*deficient (*Atf7* ^*−/−*^) male and female mice were mated, and pregnant mice received daily intraperitoneal injections of 20 μg·kg^−1^ of TNF‐α from E2.5 to E18.5. Telomere shortening was not observed in any of the tissues examined in 1‐week‐old *Atf7* ^*−/−*^ offspring (Fig. 3A). These results indicate that the *in utero* TNF‐α‐induced telomere shortening in the bone marrow, spleen, and lungs of 1‐week‐old mice was dependent on ATF7.

We then tested whether the *in utero* TNF‐α‐induced telomere shortening in the blood cells of young adult mice was dependent on ATF7. Telomere shortening was observed in the blood cells of WT but not *Atf7* ^*−/−*^ 3‐week‐old mice (Fig. 3B), indicating that the *in utero* TNF‐α‐induced telomere shortening in blood cells of young adult mice was ATF7‐dependent. Taken together, these results indicated that the *in utero* TNF‐α‐induced telomere shortening in infant and young adult mice was mediated by ATF7.

### *In utero* TNF‐α treatment induces a release of ATF7 in adult splenocytes

**Figure 4:**
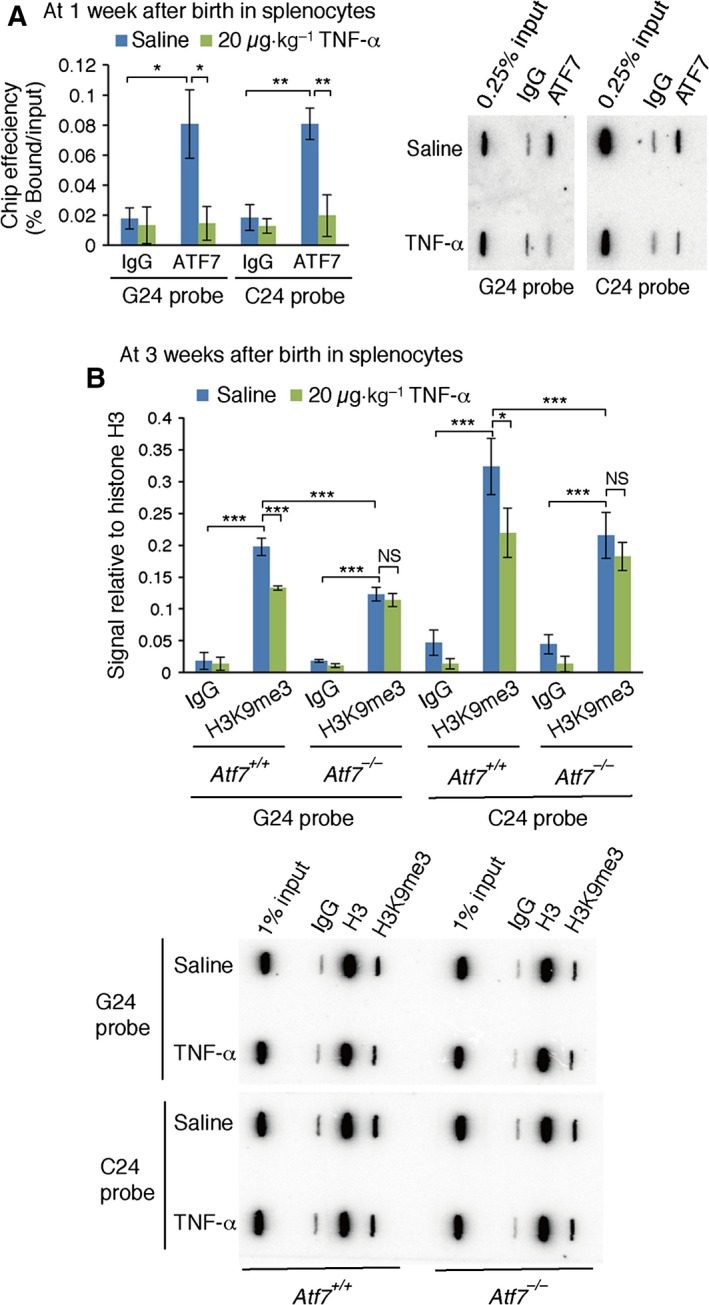
*In utero* TNF‐α treatment induces the release of ATF7 and decreases histone H3K9me3 in adult splenocytes. (A and B) WT pregnant mice were intraperitoneally injected with TNF‐α (20 μg·kg^−1^ weight) daily from E2.5 to E18.5. Splenocytes were prepared from 1‐week‐old mice (*n* = 3), and used for chromatin immunoprecipitation with anti‐ATF7 or IgG (A). Splenocytes were prepared from 3‐week‐old mice (*n* = 4), and used for chromatin immunoprecipitation with anti‐histone H3K9me3 or IgG. (B) Recovered DNA was subjected to slot‐blot hybridization with a ^32^P‐labelled telomere probe. Average signals are shown ± SD, and typical data from slot‐blot hybridization are shown below. *P <* *0.05; **P <* *0.01; ***P < 0.001; N.S., not significant.

TNF‐α‐induced ATF7 phosphorylation by p38 causes a release of ATF7 and Suv39 h1 histone H3K9 trimethyltransferase from telomeres in HeLa cells (T. Maekawa, B. Liu & S. Ishii, unpublished). Therefore, we examined whether TNF‐α treatment during E2.5–E18.5 induced the release of ATF7 from telomeres in splenocytes of 1‐week‐old mice. Splenocytes were prepared from 1‐week‐old mice exposed to 20 μg·kg^−1^ TNF‐α or saline from E2.5 to E18.5, and the chromatin was crosslinked, fragmented and immunoprecipitated using an anti‐ATF7 monoclonal antibody. Immunoprecipitated DNA was hybridized with a telomere G or C probe. The results indicated that *in utero* TNF‐α treatment reduced the amount of ATF7 on the telomeres of splenocytes from 1‐week‐old mice (Fig. 4A), suggesting that TNF‐α induces the irreversible release of ATF7 from telomeres.

We also examined whether TNF‐α treatment between E2.5 and E18.5 induces a decrease in histone H3K9 trimethylation (H3K9me3) in the splenocytes of 3‐week‐old mice. The results of similar ChIP/Slot‐blot hybridization experiments using anti‐H3K9me3 indicated that *in utero* TNF‐α treatment reduced the level of H3K9me3 on the telomeres of splenocytes from 3‐week‐old mice (Fig. 4B).

## Discussion

An epidemiological study indicated that exposure to stress during intrauterine life is associated with shorter telomere length in young adulthood 10. In the present empirical study, injection of TNF‐α into pregnant mice caused telomere shortening in young adult mice, supporting the results of previous epidemiological studies. Furthermore, telomere shortening occurred in an ATF7‐dependent manner, suggesting that TNF‐α induces ATF7 phosphorylation via p38, which leads to telomere shortening. Telomere shortening was maintained in young adult mice. *In utero* TNF‐α treatment‐induced telomere shortening was observed only in blood cells in 3‐week‐old mice, indicating that telomere shortening was retained for approximately 1 month. The TNF receptor, p38, and ATF7, which are involved in TNF‐α‐induced and ATF7‐dependent telomere shortening, are not only expressed in blood cells, but also in other tissues, indicating that this specificity is not due to tissue‐specific expression of these factors. The mechanisms underlying the tissue specificity of telomere shortening remain unclear; however, the fact that the lifespan of blood cells is approximately 1 month after differentiation from hematopoietic stem cells could be important.

*In utero* TNF‐α treatment‐induced telomere shortening detected at 3 weeks after birth was lost at 6 weeks after birth in tissues and blood cells. TNF‐α induces ATF7 phosphorylation by p38, which may cause telomere shortening. On the other hand, TNF‐α induces NF‐κB p65 nuclear translocation, which promotes the translocation of telomerase from the cytosol to the nucleus, leading to telomere lengthening 24. Furthermore, TNF‐α‐induced ATF7 phosphorylation causes the release of Suv39 h1 histone H3K9 trimethyltransferase, similar to ESET on the serotonin receptor 5b gene and G9a on innate immune genes. In the present study, *in utero* TNF‐α treatment reduced the level of H3K9me3 on the telomeres of splenocytes from 3‐week‐old mice. Given that Suv39 h1/2 mutations induce telomere lengthening 25, the TNF‐α‐induced decrease in H3K9me3 on telomeres may cause telomere lengthening in a similar manner. TNF‐α may shorten or lengthen telomeres depending on the balance between different processes. Nevertheless, the role of ATF7 in stress‐induced telomere shortening remains unknown. The expression of NF‐κB p65, Suv39 h1 and other factors that regulate the levels of histone H3K9me3 may change during development, and such changes could result in the loss of *in utero* TNF‐α‐induced telomere shortening, as observed at 6 weeks after birth in this study.

The results of an epidemiological study indicating that stress exposure in intrauterine life is associated with shorter telomere length in young adulthood 10 were based on the analysis of blood cells. The results of present study show that telomere shortening occurs in specific tissues and cells, suggesting that such telomere shortening is not necessarily linked to a short lifespan, although telomere length is widely believed to be correlated with lifespan 3. Telomere shortening in specific tissues could have an effect on metabolism and increase the level of reactive oxygen species (ROS), as reported previously 5. As diffusible molecules, ROS may be toxic to other tissues and result in the development of certain diseases.

In summary, we found that *in utero* TNF‐α treatment‐induced telomere shortening in specific tissues of infant and young adult mice in an ATF7‐dependent manner. The present findings may improve our understanding of the mechanism of stress‐induced telomere‐related human diseases.

## Author contributions

BL, TM and SI conceived and designed the experiments. BL and TM performed the experiments. BL, TM and SI analysed the data, and wrote the manuscript.

## Supporting information


**Table S1.** Sequences of oligonucleotides and primers used.
