# Supplementary material for: In utero TNF‐α treatment induces telomere shortening in young adult mice in an ATF7‐dependent manner
Source: FEBS Open Bio. 2016 Jan 4;6(1):56–63. doi: 10.1002/2211-5463.12006 (PMC4794794; doi:10.1002/2211-5463.12006)
Supplement: Supplementary file 1 — Table S1. Sequences of oligonucleotides and primers used. [file FEB4-6-56-s001.docx]

**Table S1. Sequences of oligonucleotides and primers used**

| **Q-PCR primer** | | | |
| --- | --- | --- | --- |
| Gene or region | Forward | | Reverse |
| Telomere | GGTTTTTGAGGGTGAGGGTGA  GGGTGAGGGTGAGGGT | | TCCCGACTATCCCTATCCCT  ATCCCTATCCCTATCCCTA |
| Mouse 36B4 | | ACTGGTCTAGGACCCGAGAAG | TCAATGGTGCCTCTGGAGATT |
